# Radiographic assessment of sacral hiatus anatomy with backache

**DOI:** 10.12669/pjms.39.6.7112

**Published:** 2023

**Authors:** Samia Khalid Khokhar, Ambreen Surti, Aisha Qamar, Saneed Khaliq

**Affiliations:** 1 Dr. Samia Khalid Khokhar, MPhil, Department of Anatomy, Bahria University Health Sciences, Karachi, Pakistan; 2 Dr. Ambreen Surti, MPhil, Department of Anatomy, Bahria University Health Sciences, Karachi, Pakistan; 3 Prof. Dr. Aisha Qamar, MPhil, Department of Anatomy, Bahria University Health Sciences, Karachi, Pakistan; 4 Dr. Saneed Khaliq, MPhil, Department of Anatomy, Mekran Medical College, Turbat, Balochistan Pakistan

**Keywords:** Radiographic assessment, Sacral hiatus, Anatomical variations, Backache

## Abstract

**Objectives:**

To determine hiatal variations in cases of backache and controls on radiographs and association of age groups with hiatal parameters in patients with backache versus normal individuals.

**Methods:**

This case control study on 178 patients (89 cases and 89 controls), aged from 18-65 years, selected by non-probability convenience sampling was conducted at Radiology Department of PNS Shifa Hospital, Karachi over six months.The sacral hiatus was identified on lumbosacral spine radiographs. Both metric and non-metric parameters of hiatus with respect to sacral vertebra were noted and compared between the groups.

**Results:**

Inverted “U” was the most common type observed in cases with base of hiatus at S5 level. Comparison of hiatal shape among different age groups showed inverted “U” and inverted “V” types among all age groups. Hiatal anteroposterior diameter and width were greatest in 36-45-year age group, and it was longest in 46-55-year age group. Determination of relationship between sacral hiatal parameters and incidence of low back pain showed positive association of inverted “U” and “M” shapes with back pain. Increased risk of back pain was observed with high apex (first sacral vertebra (S1)).

**Conclusion:**

Strong positive correlation was determined with inverted “U” and “M” shapes, and level of apex at S1 with low backache.

## INTRODUCTION

The sacrum is a wedge-shaped bone consisting of five fused sacral vertebrae. It is articulated with lumbar vertebrae superiorly, coccyx inferiorly and iliac bones laterally.^1^ Sacral hiatus is a midline space on dorsal surface of sacrum, formed by non-union of lamina of fifth and (less commonly) fourth sacral vertebrae.^2^ Sacral cornua (horn) are important landmarks to identify the hiatus for procedures like caudal epidural anesthesia and is formed by downward projection of inferior articular process of fifth sacral vertebra. The hiatus can be identified on body surface two inches beneath skin of natal cleft above the tip.^3^ Sacral hiatus is covered by superficial and deep posterior coccygeal ligaments, superficial being attached to margins of hiatus and deep to floor of hiatus respectively. It contains fifth pair of sacral nerve roots, coccygeal nerve roots, filum terminale and fibro-fatty tissue. Anomalies of the sacrum are heterogenous. Defects may be minor, incidentally discovered on radiographs. They have been linked to the ossification of the sacrum which is usually completed by the fourth decade of life. 4

The hiatus is frequently used for injections and catheterization in regional anesthesia and analgesia in pregnant women, children, and adults.^5^ An accurate knowledge of anatomical variations of sacral hiatus is necessary for a successful procedure through it.^3^ It is crucial to correctly identify apex and antero-posterior diameter of hiatus for this purpose. Safe option for entrance into hiatus is through its base. This approach has also been used in anorectal surgeries with regional anesthesia. Success rate of caudal epidural anesthesia increases with experience of surgeon and significantly increases pain reduction. Sacral hiatus variations have also been seen in cases of non-specific low back pain due to reduced attachment area for deep muscles of the back. 6

Limited data is found in literature with regards to anatomical variations of sacral hiatus in our population. So, this study aimed to bridge the gap in knowledge regarding hiatal variations by radiological study in our population and to determine association of hiatal parameters with backache and age in cases and controls. The observations of this study will be beneficial for clinical practitioners in orthopedic surgery, and anesthesia for treatment of chronic pain, rehabilitation medicine, subumbilical surgeries and complication free caudal anesthesia in adults.

## METHODS

This is a case control study which included 178 subjects (89 cases and 89 controls). Sample size was calculated using Open Epi version 3.0, open source calculator, with 95% confidence interval. Participants were selected by non-probability convenience sampling. This study was conducted at Radiology Department of PNS Shifa Hospital, Karachi from January till June 2020 (6 months), after obtaining ethical approval from ERC of Bahria University Health Sciences Campus, Karachi (ERC 03/2020).

### Inclusion and Exclusion Criteria:

Male and female patients, 18-65 years of age, diagnosed with acute and chronic non-specific low back pain clinically examined and referred from outpatient departments of PNS Shifa were included in the study as cases. Asymptomatic males and females between ages of 18-65 years, referred to the radiology department for evaluation of other conditions were enrolled as controls. The exclusion criteria for both groups was as follows: pregnancy, previous history of sacral fracture, spinal injury, back trauma, rheumatoid arthritis, osteoarthritis, ankylosing spondylitis, transitional vertebra, diffuse skeletal hyperostosis, disc herniation, reduced disc space, transitional lumbosacral vertebrae, or any degenerative changes on radiographs due to age. The patients in the cases group were further divided into five groups according to age.

### Imaging Technique:

Images of lumbosacral spine were taken in lateral and anteroposterior views by means of Toshiba Rotanode TM Medical X-Ray Machine and images were transferred to Agfa Fuji Pacs System. CR system being automatic with reusable plates generated images at a high kilovoltage peak but with lower milliampere second thereby reducing radiation dose in patients.

### Parameters Used:

Both metric (length, transverse width and anteroposterior diameter of sacral hiatus) and non-metric parameters like shape of hiatus, and level of base with respect to sacral vertebrae were noted in both anteroposterior and lateral view of the images. These parameters were compared between cases and controls.

The apex and base of the hiatus was determined with reference to the sacral vertebra, the apex being the highest point and base the lowest point. The length of the hiatus was determined by the distance between the apex and center point of the base. The width was measured between the sacral cornua. The depth was measured in the lateral view as the distance between the apex and posterior wall of sacral canal. The shapes of the hiatus were determined based on appearance of the hiatus on imaging as well as the margins of the hiatal opening.^3^,^5^

### Statistical Analysis:

All noted data was entered and analyzed on SPSS version 23. Frequency was used to depict non-metric data while Fischer’s Exact test and One Way ANOVA were used to measure association between categorical variables and quantitative values with p-value of ≤0.05 taken as significant.

## RESULTS

Six different shapes, inverted “U”, inverted “V”, “M” shape, “dumbbell”, “irregular” and “bifid”, of sacral hiatus were noted in our study radiographically. As shown in Table-I, inverted “U” was most common type observed in cases while inverted “V” was most commonly observed in controls. However, in controls inverted “V” was found as most common type (50.6%) followed by inverted “U” (32.6%). While “M” shape was least common type (2.2%) determined in controls of present study.

**Table-I:** Frequency and percentage of Sacral Hiatus Shapes in Cases.

	Cases						Controls					

	Age					P-value	Age					P-value
Shape of Hiatus	18-25	26-35	36-45	46-55	56-65	0.851	18-25	26-35	36-45	46-55	56-65	0.174
Inverted U	4	13	14	12	4		4	7	10	4	4	
	44.4%	52.0%	51.9%	54.5%	66.7%		19.0%	36.8%	38.5%	28.6%	44.4%	
Inverted V	3	7	9	5	1		15	10	11	4	5	
	33.3%	28.0%	33.3%	22.7%	16.7%		71.4%	52.6%	42.3%	28.6%	55.6%	
M shape	0	1	0	2	1		0	0	2	0	0	
	0.0%	4.0%	0.0%	9.1%	16.7%		0.0%	0.0%	7.7%	0.0%	0.0%	
Dumbbell	1	2	1	1	0		1	1	2	1	0	
	11.1%	8.0%	3.7%	4.5%	0.0%		4.8%	5.3%	7.7%	7.1%	0.0%	
Bifid	1	0	2	0	0		0	1	1	1	0	
	11.1%	0.0%	7.4%	0.0%	0.0%		0.0%	5.3%	3.8%	7.1%	0.0%	
Irregular	0	2	1	2	0		1	0	0	4	0	
	0.0%	8.0%	3.7%	9.1%	0.0%		4.8%	0.0%	0.0%	28.6%	0.0%	
Total	9	25	27	22	6		21	19	26	14	9	
	100.0%	100.0%	100.0%	100.0%	100.0%		100.0%	100.0%	100.0%	100.0%	100.0%	

To compare the level hiatal base between cases and controls, Fisher’s Exact test was applied. The base was most often found at S5 level and least commonly observed at level of coccyx in cases (Table-II). However, difference between cases and controls was not statistically significant (Table-II).

**Table-II:** Comparison of Level of Base of Sacral Hiatus between Cases and Controls.

Hiatal Base	Case (n=89)	Control (n=89)	Total	p-value Fischer Exact
S3	4	3	7	0.892
	4.5%	3.4%	3.9%	
S4	24	20	44	
	27.0%	22.5%	24.7%	
S5	59	64	123	
	66.3%	71.9%	69.1%	
Coccyx	2	2	4	
	2.2%	2.2%	2.2%	

Fisher’s exact test was used to compare hiatal shape among different age groups of cases. For this purpose, cases were further divided into five groups (18-25, 26-35, 36-45, 46-55 and 56-65) according to age (year). Inverted “U” (52.8%) and inverted “V” (28.1%) were the most common types among all age groups. “M” shape was however observed in only age groups of 46-55-year and 56-65-year. “Dumbbell” and “bifid” shapes were not observed in older age groups (46-55 and 56-65 year). However, result was statistically insignificant (p-value = 0.886).

Binary logistic regression analysis was done to observe relationship between sacral hiatal parameters and incidence of low back pain in cases. It was noted that inverted “U” (OR 1.6) and “M” shapes (OR 2.0) which were commonly observed in older age groups, showed a positive association with back pain. Increased risk of developing back pain was observed if level of apex was at S1 level (OR 2.5) as shown in Table-III.

**Table-III:** Relationship between Sacral Hiatal Parameters and Low Back Pain Binary.

Hiatal Shape	Odds Ratio	95% C.I.	p-value
Inverted U	1.621	0.432 - 6.086	0.474
Inverted V	.556	0.147 - 2.106	0.387
M shape	2.000	0.244 - 16.362	0.518
Dumbbell	1.000	0.173 - 5.772	0.999
Bifid	1.000	0.132 - 7.570	0.999
Irregular	1		
***Hiatal Apex***			
S1	2.5	0.194 - 32.194	0.482
S2	1.713	0.460 - 6.372	0.422
S3	0.551	0.158 - 0.920	0.35
S4	1		
***Hiatal Base***			
S3	1.33	0.113 - 15.704	0.819
S4	1.2	0.155 - 9.301	0.861
S5	0.92	0.126 - 6.755	0.936
Coccyx	1		

Logistic Regression Analysis in cases (n=89).

In this study age of participants were compared with metric hiatal parameters. Each metric parameter of sacral hiatus was analyzed for each age group to determine any significant difference in hiatal anatomy with age (Table-IV). Longest average hiatal length was observed in age groups 46-55 year (31.79±10.42mm) and 36-45 year (31.59±10.88mm). The shortest average hiatal length was recorded in 18-25 years (23.15±3.53mm) age group. The difference in mean length of sacral hiatus amongst different age groups was not statistically significant (Table-IV). The average anteroposterior diameter of sacral hiatus was largest in 36–45 years (3.91±3.23mm) and smallest in 18-25 years (2.98±1.23mm) age groups. There was no statistically significant difference between measurements of anteroposterior diameter with regards to age in cases (Table-IV). The widest sacral hiatus was observed in the backache cases in the 36-45 years (14.04±4.93mm) and 46-55 year (13.85±5.39mm) age groups. In the normal individuals the widest sacral hiatus was also detected in the 46-55 year (13.12±13.18mm) age group. In the 36-45 year age group the transverse width was smaller (10.99±3.46mm) as compared to cases. The smallest value was present in 18-25 years (12.25±1.80mm) age group in both cases and controls. The difference in transverse widths between age groups was not statistically significant (Table-IV).

**Table-IV:** Comparison of Age with Metric Hiatal Parameters (n=178).

		Case				Control			

Variables	Age	n	Mean	Std. Deviation	P-value	n	Mean	Std. Deviation	P-value
Hiatal length in mm	18-25	9	23.15	3.53	0.23	21	23.22	6.55	0.202
	26-35	25	29.45	10.69		19	25.85	11.65	
	36-45	27	31.59	10.88		26	21.53	5.59	
	46-55	21	31.79	10.42		14	26.40	5.65	
	56-65	6	30.43	4.06		9	22.06	4.92	
	Total	88	30.09	10.02		89	23.67	7.56	
Antero-posterior Diameter	18-25	9	2.98	1.23	0.567	21	2.83	0.87	0.7
	26-35	25	3.26	1.21		19	3.19	1.67	
	36-45	27	3.91	3.23		26	3.25	1.45	
	46-55	21	3.18	1.22		14	3.11	1.16	
	56-65	6	2.76	1.09		9	2.69	0.75	
	Total	88	3.38	2.05		89	3.06	1.28	
Transverse Width	18-25	9	12.25	1.80	0.361	21	11.33	3.01	0.29
	26-35	25	13.22	3.31		19	11.76	4.30	
	36-45	27	14.07	4.93		26	10.99	3.46	
	46-55	21	13.85	5.39		14	13.12	3.48	
	56-65	6	10.41	3.07		9	13.11	2.48	
	Total	88	13.34	4.33		89	11.78	3.51	

## DISCUSSION

The development of sacrum takes place by fusion of 1^st^-5^th^ sacral vertebrae. Each half of vertebral arch forms complete sacral canal by fusing posteriorly. However, posterior non-fusion of lamina of fifth sacral vertebra results in formation of sacral hiatus.^7^ Thus, sacrum displays numerous variations particularly in this area. The extent, shape, length, width and anteroposterior diameter of hiatus depends on number of laminae which fail to fuse posteriorly.^8^ Knowledge of these parameters is essential for accurate localization of hiatus for successful administration of caudal epidural block.^9^

These parameters are unique in various regions of world and different races.^10^ So, this study was planned to undertake radiological assessment of sacral hiatus and to compare findings among cases of non-specific low backache with healthy subjects. The results of present study revealed that inverted “U” was most common shape among cases of backache, followed by inverted “V”. This result was in accordance to Bagoji et a1^1^ who also observed inverted “U” shape as most frequent, followed by inverted “V” in dry sacral bones and in AP radiographs of lumbosacral spine.

The results were also similar to another study^11^ by Punja et al that also found inverted “U” as most common shape of hiatus in dry human sacra. However, our results were contradictory to an Ethiopian study done on dry human sacra^12^ and study by Bayat & Khosrobeigi 13 on dried Iranian sacra. The shape they observed most frequently were inverted “V” followed by inverted “U”. Shape of sacral hiatus is crucial because of its importance in successful administration of CEB as an irregular shaped hiatus might result in failure of such procedure.^12^

However, both these shapes (inverted U & V) are regarded as normal variants as they provide adequate space for inserting needle into sacral canal without hindrance.^14^ This difference in shape was likely due to differences in genetic make-up, health, weight, height and built among different races. Iranians are generally taller and heavy built as compared to Pakistani population which is the most likely reason for this disagreement. Similar to our study results, Javed et al also found inverted “U” as most common shape in dry Pakistani sacral bones.^15^

In the present study, base of sacral hiatus was most found at level of 5^th^ sacral vertebra. This was similar to many studies^16^-^18^ which also found hiatal base at level of 5^th^ sacral vertebra in 98.6%, 75.19% and 89.03% of dry human sacral bones. However, the present study did not find statistically significant difference in level of base between cases and controls. Similar observation was made by another study conducted on 100 patients in 2019.^19^

In the current study, frequency of different hiatal shapes was also compared with age groups of cases as parameter tend to vary with age.(reference) For this intention, cases were segregated into five groups as mentioned in methods. Similar groups were also highlighted in study by Baske & Mondal .^19^ Inverted “U” and inverted “V” were most observed types among all age groups. Findings similar to our study were observed by Joshi.^20^

The results of present study revealed a positive correlation between backache and inverted “U” and “M” shapes. Equal risk was also observed for dumbbell and bifid hiatal shapes. Correlation of inverted “V” shape with low back pain was not observed. The study also revealed the greatest risk of low back pain when hiatal apex was observed at level of S1. Our study found three (3.4%) cases of low backache with apex at level of S1 as compared to one (1.1%) in control. Apex at level of S1 was reported in 1.1% cases by Bagheri & Govsa 10 and 2% sacra by Geeta & Bidarkotimath .^21^ It has been suggested that variations in development of sacral hiatus can cause decreased area for extensor muscle attachment at back resulting in painful conditions. A mild stress causes these extensor muscles to strain more resulting in severe backache. Higher the level of apex, more will be the chances for developing backache.^22^

The present study also showed that risk of backache was correlated with base at levels S3 and S4. Base levels at S5 revealed no significant risk of low back pain. However, this result is contradictory to Mondal & Baske 23 who determined that 6% of cases of backache had hiatal base at S4 and above, whereas 94% of study subjects revealed hiatal base at S5.

In the present study, hiatal metric parameters within cases of backache were also evaluated for variations related to age. Results showed longer hiatal length in cases with age groups of 36-45 and 46-55 year as compared to 26-35- and 56-65-year age groups. Mondal & Baske 22 found similar results. Length of sacral hiatus was more in age group of 30-40 year, as compared to younger or older age groups. Our results were also similar to Hussain et al 24 who found highest incidence of backache in shopkeepers among age group of 31-38 year as compared to younger or older age groups in Lahore.^24^ In current study hiatal width was greatest in age group 36-45 year while lowest value was seen in age group of 56-65 year. The results of our study were similar to another study^25^ that found transverse width of sacral hiatus from 11-15mm in most of dry sacral bones examined.

The anteroposterior diameter of sacral hiatus in current study was greatest in 36-45-year age group while lowest value was observed in 56-65-year age group. Elumalai et al^7^ observed anteroposterior diameter of sacral hiatus ranging from 3.7mm to 15mm in cases of low backache whereas their values ranged from 4mm-8mm in healthy controls. These differences were most likely because those patients are Nepali and each race has differences in development and fusion of sacral bone as mentioned in literature.^8^,^12^,^22^

### Limitations:

It is a single center and a small sample size was used due to time constraints.

## CONCLUSION

Inverted “U” was most commonly observed hiatal shape in cases as compared to inverted “V” in controls. Inverted “U” and “M” shapes exhibited strong correlation with backache most commonly observed in older age groups. When correlated with symptoms of backache, there was strong positive correlation with inverted “U” and “M” shapes, which were commonly observed in older age groups and level of apex at S1.

### Recommendation:

Studies using detailed imaging techniques such as CT scan and MRI should be conducted in a larger population to further assess hiatal variations.

### Authors’ Contribution:

**SKK:** research concept, design, data collection, responsible for integrity and accuracy of work.

**ASK & AQ:** writeup & statistical analysis.

**SK**: editing & final review of manuscript.
